# Combined citicoline and Cerebrolysin for neuroprotection in traumatic brain injury: a retrospective cohort analysis

**DOI:** 10.3389/fneur.2025.1684981

**Published:** 2025-12-02

**Authors:** Philipp Schlager, Ivan Grgac, Guenther Herzer, Helmut Trimmel

**Affiliations:** 1 Department of Anesthesia, Emergency and Intensive Medicine, University Hospital of Wiener Neustadt, Wiener Neustadt, Austria; 2 Danube Private University – Department of Medicine, University Hospital of Wiener Neustadt, Wiener Neustadt, Austria; 3 Karl Landsteiner Institute of Emergency Medicine, University Hospital of Wiener Neustadt, Wiener Neustadt, Austria

**Keywords:** sTBI, neuroprotection, Cerebrolysin, citicoline, Glasgow Outcome Scale Extended

## Abstract

**Introduction:**

Severe traumatic brain injury (sTBI) remains a major cause of long-term disability and mortality worldwide. Beyond the initial mechanical damage, a cascade of secondary injuries involving neuroinflammation, oxidative stress, and excitotoxicity exacerbates neural dysfunction. Neuroprotective agents such as citicoline and Cerebrolysin have shown promise in addressing these complex mechanisms and supporting recovery. This study aimed to evaluate whether a combination therapy of citicoline and Cerebrolysin improves neurological outcomes compared to citicoline monotherapy in patients suffering from sTBI.

**Methods:**

A retrospective cohort analysis was conducted at a single university hospital. Patients with sTBI treated between 2012 and 2021 were included. Two cohorts were matched based on a validated prognostic scoring system to ensure comparability. One group received citicoline monotherapy, while the other received a combination of citicoline and Cerebrolysin. The primary endpoint was neurological function 6 months after injury. Secondary outcomes included survival and duration of stay in the intensive care unit and hospital.

**Results:**

Eighty patients were analyzed. While there was no statistically significant difference between the two groups in neurological function or mortality at 6 months, patients receiving the combination therapy showed a tendency toward better neurological outcomes. Notably, this group also exhibited more severe baseline injury profiles, which may have influenced the results.

**Conclusion:**

Combined treatment with citicoline and Cerebrolysin may offer additional benefits for neurological recovery in patients with severe traumatic brain injury. Although statistical significance was not reached, the observed trend supports the need for further prospective, controlled studies to explore potential therapeutic advantages.

## Introduction

Severe traumatic brain injury (sTBI) is a global epidemic and represents both an individual and socioeconomic burden (1–3). In addition to permanent motor impairments, essential cognitive and emotional functions are frequently affected. Mechanical damage to the brain tissue leads to the release of neurotoxic molecules (“damage-associated molecular patterns,” DAMPs) (4) and coagulation-active substances (tissue plasminogen activator tPA, and fibrinogen) that affect the integrity of the blood–brain barrier (BBB). The release of excitotoxic amino acids, especially glutamate, leads to excessive stimulation of their corresponding receptors [in particular N-methyl-D-aspartate (NMDA) receptors] (5), resulting in increased intracellular calcium ions (Ca^++^) concentrations. This, in turn, triggers the activation of intracellular autolytic enzymes (proteases and phosphatases), mitochondrial dysfunction, and the production of reactive oxygen species (ROS). The inflammatory response that follows BBB disruption (neuroinflammation) (6), together with ROS-induced lipid peroxidation (7, 8), cytotoxic, and vasogenic edema, and massive impairment of cell metabolism, can persist for days or even weeks, significantly aggravating the primary mechanical injury (7, 9–12).

Endogenous paracrine factors enable the brain to exhibit a certain degree of resistance to post-traumatic secondary damage. In the 1950s, the first neurotrophic factor (NTF), nerve growth factor (NGF), was discovered (13, 14). Neurotrophic peptides, dimeric protein complexes, can trigger cell proliferation, synaptogenesis, migration, and regeneration of brain cells in specific regions. NTFs include brain-derived neurotrophic factor (BDNF), hypoxia-inducible factor (HIF)-1, and vascular endothelial growth factor (VEGF) (15).

The concept of administering neurotrophic drugs is based on these pathophysiological mechanisms and aims to utilize these endogenous mechanisms to reduce secondary noxious stimuli while supporting vascular and axonal regeneration and promoting neuroplasticity. Over the past three decades, a wide range of neuroprotective and regenerative approaches has been investigated in both research and clinical practice (16–18).

Citicoline has been used for this indication in our intensive care unit for more than a decade (19–21). This drug, also known as cytidine-5′-diphosphocholine (CDP-choline), plays a key role in the synthesis of phosphatidylcholine, an important component of cell membranes. After oral or intravenous administration, citicoline can cross the BBB in the form of its hydrolyzed components (choline and cytidine triphosphate). Citicoline plays an important role in modulating membrane structure and function, influences neurotransmission, and exerts a protective effect on nerve cells. Increased availability of choline activates the biosynthesis of structural phospholipids, supporting the integrity and repair of neuronal and glial cell membranes. Additionally, citicoline contributes to the synthesis of the neurotransmitter acetylcholine (21–23). Additionally, citicoline can inhibit the activity of phospholipases. It has also been linked to the promotion of glutathione synthesis, an important antioxidant that contributes to the reduction of oxidative stress and lipid peroxidation (24). In addition to animal data, there are a number of clinical studies that have investigated its effectiveness in the treatment of stroke sequelae, cognitive impairment, and traumatic brain injury (20). A recent meta-analysis investigating the effectiveness of citicoline in patients with TBI showed that citicoline significantly improved patient independence (21).

Cerebrolysin has also been used for decades to treat neurological disorders. It is derived from highly purified (pig) brain proteins through standardized enzymatic degradation and consists of 25% low-molecular-weight peptides and free amino acids (25, 26). Several fragments of NTFs have been identified using immunoassay (ELISA). These stimulate neurotrophic signaling pathways and thus promote the growth, differentiation, and survival of nerve cells (27–29), as well as angiogenesis, dendritic proliferation, axonal sprouting, myelination, and remodeling of the neurovascular unit (29, 30). In addition to the neurovascular unit (endothelial cells, pericytes, and extracellular matrix), target structures include neurons, microglia cells, oligodendrocytes, astrocytes, and their myelin sheaths. Cerebrolysin has antioxidant properties and improves energy metabolism by supporting mitochondrial function and increasing ATP production (15, 29). Clinically, an improvement in cognitive performance has been demonstrated in patients with moderate and severe TBI. Meta-analyses with a total of more than 10,000 participants have shown that intravenous administration of Cerebrolysin significantly improves the extended Glasgow Outcome Score (GOSE) and the modified Rankin Scale (mRS) (31–33). Data from the CAPTAIN studies further demonstrate that Cerebrolysin can significantly improve cognitive function, reduce neurological deficits and post-traumatic depression, and promote functional recovery (34–37). Since July 2018, we have supplemented adjuvant therapy with citicoline in patients with sTBI by administering Cerebrolysin at our institution.

However, limited data are available on the combined administration of the two substances. An *in vitro* study showed that both drugs increase BDNF expression, which indicates an improvement in cellular defense mechanisms (38). In a prospective clinical study involving 60 patients with mild to moderate TBI, Cerebrolysin was investigated in combination with citicoline compared to citicoline alone in patients with moderate TBI (mTBI). Patients receiving combination therapy tended to have better recovery rates (39). Case reports of severely polytraumatized patients with sTBI have also reported surprisingly positive neurological outcomes following combination therapy with Cerebrolysin and citicoline (40). In the present study, we investigated the two therapeutic strategies using a retrospective analysis: treatment with citicoline and the combined administration of citicoline and Cerebrolysin.

## Materials and methods

**Figure 1:**
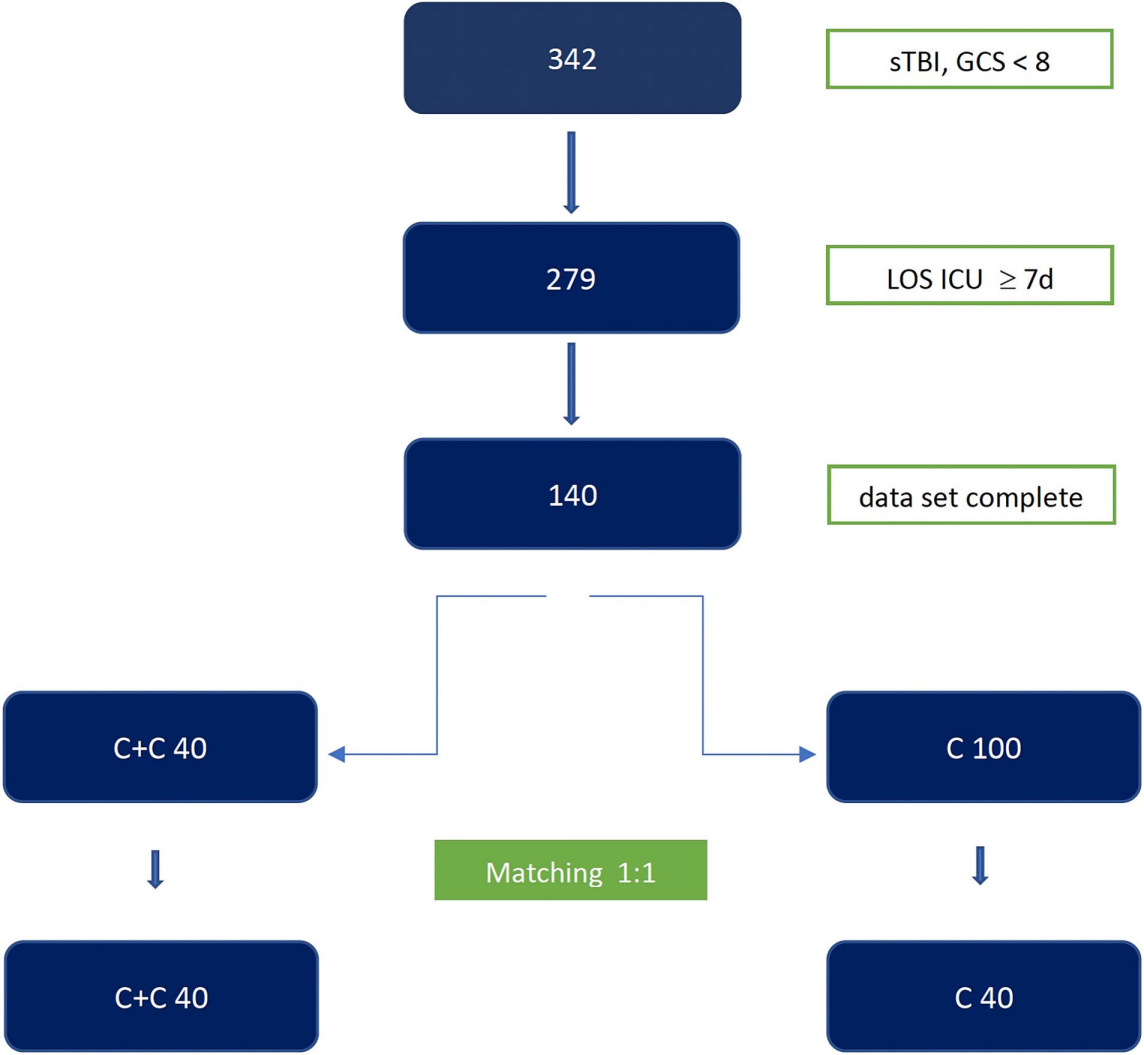
Patient flowchart. sTBI, severe traumatic brain injury; GCS, Glasgow Coma Scale; LOS ICU, length of stay in the intensive care unit; d, days; C, citicoline; C + C, citicoline + Cerebrolysin group.

With the approval of the Ethics Committee of the State of Lower Austria (EC number GS4-EK-4/828-2022), we conducted a retrospective cohort analysis of patients aged 17–99 with sTBI (initial GCS ≤ 8 at the emergency site) who were treated at the Wiener Neustadt Regional Hospital between 1 January 2012 and 31 December 2021. Different injury patterns and pre-existing comorbidities make standardized comparisons among trauma patients fundamentally difficult. We decided to use matching based on a prognostic score developed specifically for patients with traumatic brain injury (TBI-IMPACT Calculator) (41). Age and sex were also taken into account (Table 1). SOFA score, SAPS 3, main diagnosis, and cause of accident were also recorded. From a total cohort of 342 patients with sTBI (age > 16 or < 100 years) in the respective study period, two groups with the same predictive neurological outcome and survival probability could be formed (Figure 1 and Table 2a). Patients with the following conditions were excluded:

- GCS ≤ 8, but who had not received citicoline or Cerebrolysin.
- Intensive care unit (ICU) length of stay less than 7 days.
- incomplete minimum data set.
- Lack of consent (if applicable, from a legally authorized representative) or inability to conduct a telephone interview (missing data, patient deceased, or whereabouts unknown).

**Table 1:** Matching parameters.

Time	Variable	Categories/units	Type of variable	Data source
Prehospital	GCS	Points (3–15)	Ordinal	EMS recording
	GCS motor score	Points (1–6)	Ordinal	EMS recording
	Pupil reaction	Yes/No	Nominal	EMS recording
	SAP	mmHg	Metric	EMS recording
	Glucose	mg/dL	Metric	EMS recording
On admission	Age	in years	Metric	MPA
	Sex	m/f/d	Nominal	MPA
	BMI	kg/m^2^	Metric	MPA
	SAP	mmHg	Metric	PDMS
	paO_2_	mmHg	Metric	MPA
	Epidural hemorrhage	Yes/No	Nominal	MPA
	Subdural hematoma	Yes/No	Nominal	MPA
	Marshall CT score	Diffuse injury 1–4	Nominal	MPA
	Hemoglobin	g/dL	Metric	MPA

ICU, intensive care unit; MPA, medical process assistant, hospital information system; PDMS, patient data management system for intensive care medicine; GCS, Glasgow Coma Scale; paO_2_, arterial oxygen partial pressure; CT, computed tomography.

**Table 2:** (a) Predicted outcome. (b) Actual outcome.

(a)				
Variable	Group C	Group C + C	*Z*	*p*-value
	Mean ± SD Median (min/max)	Mean ± SD Median (min/max)		
Predicted 6-month mortality (%)	43 ± 22.74 36.5 (4/90)	39.45 ± 21.45 36 (4/91)	0	0.573
Predicted 6-month unfavorable outcome (%)	63 ± 22.16 64 (12/96)	60.5 ± 23.45 65 (5/97)	0	0.795

(b)				
	Group C	Group C + C	Chi or *Z*	*p*-value
6-month mortality	*n* = 13/40 (32.5%)	*n* = 12/40 (30%)	0.058	0.809
GOSE (6 months) Median (min/max)	3.46 ± 2.5 4 (1/7)	3.94 ± 2.54 4 (1/8)	0.811	0.417
mRS⁑ (6 months) Median (min/max)	3.86 ± 2.19 4 (1/6)	3.51 ± 2.09 3 (0/6)	0.598	0.550

GOSE, Glasgow Outcome Scale Extended; mRS, modified Rankin scale; max, maximum; min, minimum; SD, standard deviation.

Group C (study period 1 January 2012 to 30 June 2018) received, in addition to guideline-based treatment (39–41), citicoline at a dose of 3 g per day for 21 days by continuous intravenous infusion, which was the institutional standard for patients with sTBI at that time. The C + C group (1 July 2018 to 31 December 2021) received combination therapy consisting of citicoline (3 g per day) and Cerebrolysin (50 mL per day) according to the same regimen, which has become the new standard therapy. In the event of transfer from the ICU before day 21, therapy was discontinued in both groups at the time of transfer.

Data were collected from the Emergency Medical Service (EMS) documentation, the patient data management system (PDMS) of the ICU (ICdoc pro®, Büll Informatik GmbH, 1040 Vienna, Austria; PICIS®, Picis Clinical Solutions S.A., 08022 Barcelona, Spain), and the hospital information system (MPA®, CGM Clinical Österreich GmbH, 4400 Steyr, Austria).

The primary endpoint was the neurological outcome 6 months after the accident. This was assessed using the Glasgow Outcome Scale Extended (GOSE) with the help of a structured questionnaire administered by telephone. This questionnaire is based on the severity of the deficit; questions were asked about activities of daily living, work and leisure activities, and the ability to follow simple instructions (42).^1^ GOSE ≥ 5 was defined as “good,” and GOSE ≤ 4 as “poor.” The secondary endpoints were defined as the length of stay in the ICU or hospital and 6-month survival. The mortality rate after discharge from the hospital was determined upon request from Statistics Austria.^2^ The Chi2 test was used for two-sided significance (*α* = 5%). The odds ratio (with a two-sided 95% confidence interval) was calculated as the effect estimator.

## Results

**Figure 2:**
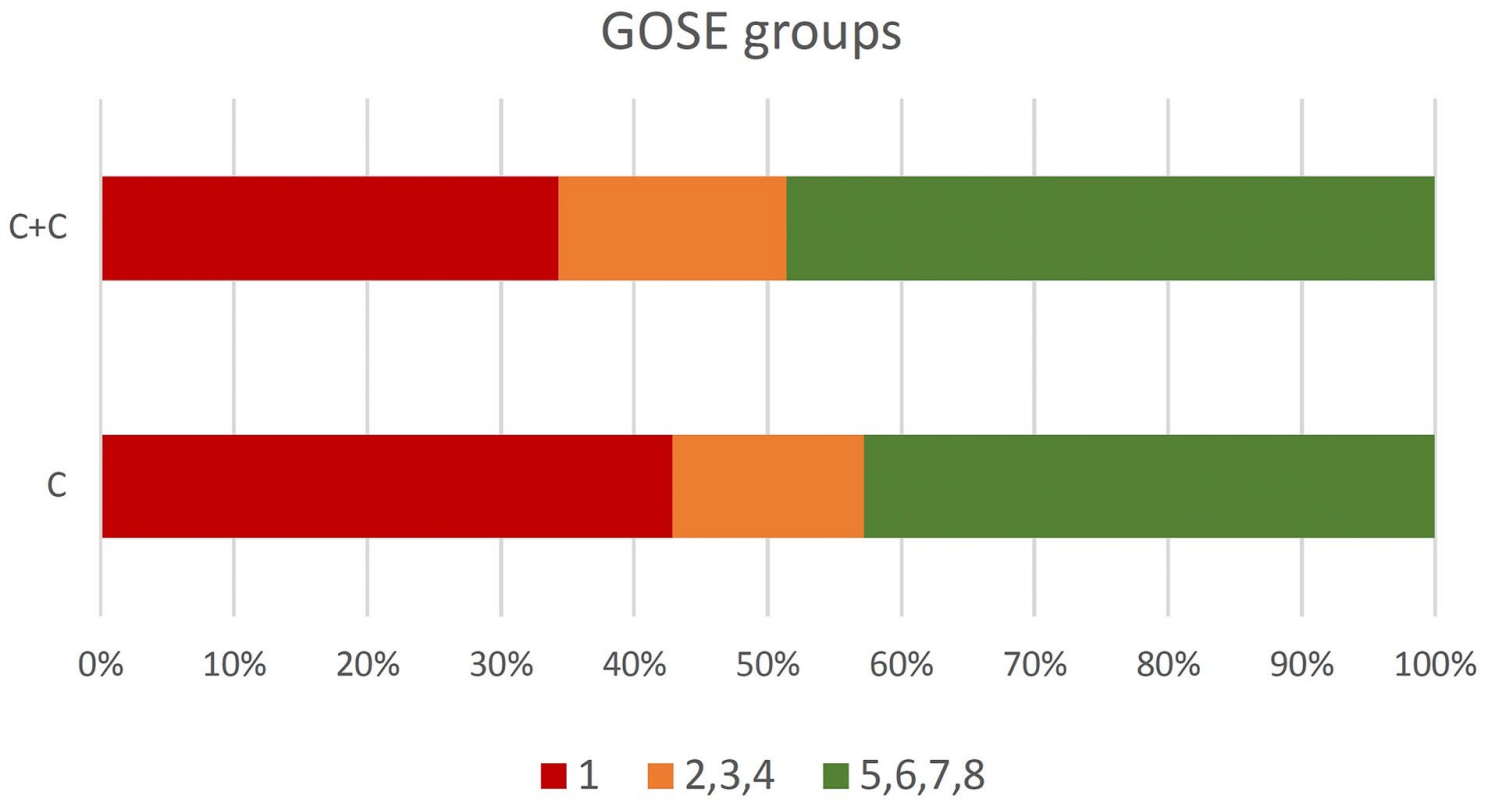
GOSE grouped. GOSE, Glasgow Outcome Score extended.

A total of 80 patients with the same predicted outcome were included (Table 2a). No difference was found in the functional neurological outcome (Table 2b). Group C achieved a mean GOSE score of 3.46 (SD ± 2.50; median 4; range 1–7), while group C + C achieved 3.94 (SD ± 2.54; median 4; range 1–8). The Mann–Whitney U-test showed no statistically significant difference in the average ranks between the groups (*Z* = 0.811; *p* = 0.417). Alternative test methods, including continuity correction (*p* = 0.630), likelihood ratio (*p* = 0.460), and linear correlation (*p* = 0.465), also confirmed the absence of a statistically significant difference. Fisher’s exact test, which is preferred for small sample sizes, also confirmed these results (two-tailed *p* = 0.610; one-tailed *p* = 0.315). Nevertheless, a trend toward better functional outcomes was evident in group C + C (Figure 2).

Furthermore, an assessment of 6-month mortality using the chi-squared test disclosed no statistically significant discrepancy between the two cohorts (Table 2b). However, both functional outcome and mortality were significantly better than predicted in both groups.

The analysis of length of stay in the ICU and the hospital did not reveal a statistically significant difference. Group C had an average length of stay of 27.28 days (SD ± 18.51; median 22 days; range 7–100 days) in the ICU, while group C + C spent an average of 32.95 days (SD ± 23.90; median 28.5 days; range 7–109 days), with a Mann–Whitney U-test result of *Z* = 0.717 and a *p*-value of 0.473. In terms of length of hospital stay, group C was 53.15 days (SD ± 31.12; median 44 days; range 23–111 days), and group C + C was 63.24 days (SD ± 37.91; median 67 days; range 13–154 days). The *Z*-value of 0.523 and a *p*-value of 0.601 show that these differences between the groups were also not significant.

## Discussion

The effectiveness of neurotropic medication is still a subject of debate; however, recent studies suggest that it is indeed efficacious. Larger studies and meta-analyses exist for citicoline and Cerebrolysin in particular, and the safety of these substances can also be considered assured (20–22, 32, 43, 44). The findings of the present study did not demonstrate any substantial disparities in the primary and secondary outcome parameters, with the exception of a modest tendency toward enhanced neurological functionality in the combination group (GOSE and/or mRS) (Figure 2). Despite meeting the matching criteria, it has to be recognized that some of the study data indicate a greater extent of injury and a more severe physiological impairment in patients in group C + C (Table 3): SAPS III and SOFA scores were higher for the C + C group, indicating greater physiological disturbances and a slightly increased risk of mortality. Comparing the results of the Marshall CT score, the assessment of diffuse injury IV was twice as common. This could indicate a more severe axonal brain injury that is not amenable to surgical intervention (45). Arterial oxygen partial pressure (paO^2^) and systolic blood pressure were also lower in group C + C upon admission. It is well known that episodes of low blood pressure have a detrimental effect on outcome (46). Additionally, the significantly longer ICU and hospital stays may indicate a more complex disease progression.

**Table 3:** Radiological and clinical findings.

Variable		Citicoline (*n* = 40)			Citicoline + Cerebrolysin (*n* = 40)		
		*n*	%		*n*	%	
Male		28	70		29	72.5	
Female		12	30		11	27.5	
		Median	IQR 0.25	IQR.75	Median	IQR 0.25	IQR.75
BMI (kg/m^2^)		25	24	28	26.27	24	29
Height (cm)		177.5	170.0	180.0	180	165.5	185.0
Weight (kg)		75.0	70.0	90.0	85	76.0	98.8
Age		55.5	37.0	68.8	58	36.25	70.0
GCS		4.5	3	6	5	3	6
GCS (motor score)		1.5	1	5	2.5	1	5
		*n*	%		*n*	%	
Hypoxia		9	22.5		9	22.5	
Hypotension		11	40		10	25	
Pupils	Both non-reactive	4	10		6	15	
	One reactive	6	15		10	40	
	Both reactive	30	75		24	60	
SAH		30	75		29	72.5	
EDH		10	25		9	22.5	
MS - Diffuse injury I		0	0		3	7.5	
MS - Diffuse injury II		14	35		15	37.5	
MS - Diffuse injury III		5	12.5		6	15	
MS - Diffuse injury IV		3	7.5		6	15	
Evacuated mass lesion		17	42.5		9	22.5	
Non-evacuated mass lesion		1	2.5		1	2.5	
Values upon admission		Median	IQR 0.25	IQR.75	Median	IQR 0.25	IQR.75
Hb (g/dL)		11.15	9.9	13.4	12.25	10.4	13.6
SAP		110	91.5	126.8	98	78.3	120.8
Glucose (mg/dL)		135.5	113.5	170.8	134.5	119.3	155.5
paO_2_ (mmHg)		131	114.3	161.8	132.5	94.0	157,8
SOFA Score		9	5	10.5	7	7	11
SAPS 3		42	30.5	60	46	20.5	62
LOS (ICU)		22	13.3	38.8	28.5	13.8	49.8
LOS (Hospital)		44	25.0	78.0	67	29.5	91.0

BMI, body mass index; IQR, interquartile range; MS, Marshall score; SAH, subarachnoid hemorrhage; EDH, epidural hematoma; ICU, intensive care unit; GCS, Glasgow Coma Scale; Hb, hemoglobin; max, maximum; min, minimum; paO_2_, arterial partial pressure of oxygen; SAP, systolic arterial pressure; SAPS 3, Simplified Acute Physiology Score 3; SOFA, Sequential Organ Failure Assessment; SD, standard deviation; LOS, length of stay.

Combined treatment with citicoline and Cerebrolysin in sTBI appears plausible from a pathophysiological perspective because the mechanisms of action are complementary—at the cellular, molecular, and systemic levels. Citicoline has a membrane-stabilizing effect and protects against excitotoxic degradation (e.g., by glutamate), while Cerebrolysin additionally promotes growth and regeneration (axons, synapses). Citicoline promotes ATP production by stabilizing mitochondrial enzymes, while Cerebrolysin can reduce oxidative stress and has a neurotrophic effect. The two substances also counteract neuroinflammation in different ways: citicoline inhibits the activation of phospholipase A2, resulting in fewer pro-inflammatory lipid mediators being generated. Cerebrolysin, on the other hand, regulates the activity of microglia and promotes neuroplasticity at the same time. In summary, the combination of citicoline plus Cerebrolysin combines substrate-based membrane and energy protection (citicoline) with neurotrophin-like signal modulation and anti-inflammatory effects (Cerebrolysin). This results in a complementary, cross-phase efficacy profile that can theoretically lead to greater functional recovery than monotherapy.

Based on recent findings, we suspect that starting the combined treatment as early as possible after trauma is essential. Early administration can mitigate secondary cell damage before irreversible cell death occurs, thereby improving the quality of the neural starting point from which regeneration proceeds. Only intravenous administration (IVA) enables immediate, controlled, and sufficient concentrations in the central nervous system—a must when minutes and hours determine the survival of nerve cells. IVA also takes advantage of the brief ‘open window’ of BBB permeability following trauma, overcomes phases of cerebral hypoperfusion through direct plasma availability, and can immediately activate neuronal membrane repair and anti-inflammatory signaling pathways. Longer administration may be essential for long-term support of the regeneration process. Some of the earlier studies showed weaknesses in this particular area (47). With regard to the combination partner Cerebrolysin, particular reference should be made to the CAPTAIN trial series, which used a much more differentiated neuropsychological test battery and showed significantly better neurological outcomes but, like us, did not demonstrate any mortality benefits (35–37, 48, 49). However, as mentioned above, the group receiving combination therapy in our study tended to have more severe injuries, which may further underscore the relevance of the trend toward a slightly higher GOSE. In summary, the results presented here support the need for further prospective, randomized studies to identify potential subgroups of patients who may benefit from combination therapy with these two neurotrophic drugs.

## Limitations

Due to the small sample size and simplified methodology, our results are by no means universally applicable. Of course, intensive care medicine is also subject to constant change over a period of 10 years, which could have a confounding effect on the outcome. The treatment of patients with sTBI in the ICU of the Wiener Neustadt Regional Hospital (now a university hospital since 2024) is strictly based on the guidelines of the Brain Trauma Foundation and the SIBICC Guidelines (50). Therefore, with the exception of the addition of Cerebrolysin to the treatment regimen, there were no changes in the department’s institutional treatment guidelines during the study period.

## Conclusion

Combined treatment with the two well-studied neuroprotective drugs citicoline and Cerebrolysin is obvious based on pathophysiological considerations. This medication indicates the possibility of supplementary benefits, as a greater number of patients in our study attained a GOSE score > 4, thereby demonstrating a higher level of independence. However, it must be noted that these results should be regarded as preliminary and do not represent conclusive evidence of efficacy. Rather, they should provide a foundation for further rigorous investigation in the form of a controlled, randomized trial. We are convinced that it is crucial to start neuroprotective or regenerative therapy as early as possible and to continue treatment throughout the entire period of active neuroinflammation, which lasts for the first few weeks after the trauma. As mentioned above, intravenous administration seems essential too, in order to avoid problems of enteral absorption when treating these often complex, multiply injured patients. A prospective, ideally multicenter and randomized “*Citolysin*” trial should comprise four groups: standard treatment vs. supplementation with the individual components discussed here vs. combination therapy.

## Data Availability

The data analyzed in this study is available on reasonable request. Enquiries regarding these data sets should be addressed to Helmut Trimmel, dr.h.trimmel@a1.net.

## Footnotes

1 https://www.center-tbi.eu/files/approved-translations/German/GERMAN_GOSE.pdf, [Supplmentary material].

2
https://www.statistik.at/
