# Supplementary material for: Combined citicoline and Cerebrolysin for neuroprotection in traumatic brain injury: a retrospective cohort analysis
Source: Front Neurol. 2025 Dec 2;16:1684981. doi: 10.3389/fneur.2025.1684981 (PMC12705382; doi:10.3389/fneur.2025.1684981)
Supplement: Supplementary file 1 [file Table_1.docx]

**Kurzleitfaden zum Telefoninterview**

**Vorstellung:**

Vorstellung. Zweck des Anrufes Studie zum Thema Schädelhirntrauma im Zusammenhang mit Intensivaufenthalt im LK Wr. Neustadt.

**Identifizierung des Patienten und Einwilligung:**

Handelt es sich um Patienten/gesetzlichen Vertreter/ anderer Angehöriger?

Gesprächspartner wird darüber aufgeklärt das Studienteilnahme absolut freiwillig und an keine Konsequenzen geknüpft ist. Patientenbezogenen Daten werden während Erstellung der Studie verarbeitet. Keine Daten, die auf den einzelnen Patienten zurückzuführen wären, werden im Fall einer Studienpublikation veröffentlicht. Es kann Beitrag am wissenschaftlichem Erkenntnisgewinn der Therapie von Schädelhirntrauma geleistet werden. Es werde nur dieses einzige, etwa 5-minütige Telefoninterview geführt werden.

**Willigt Patient/gesetzlicher Vertreter in Studienteilnahme ein?**

Falls Nein, Verabschiedung und Entschuldigung der Störung.

Falls Ja, Dokumentation von Tag und Uhrzeit der mündlichen Einwilligung des Patienten oder dessen gesetzlichen Vertreters.

**Anschließend Telefoninterview.**

**Abschluss des Gespräches:**

Offene Fragen des Gesprächspartners?

Hinterlassen einer Rückrufnummer für ev. spätere Fragen des Gesprächspartners.

Bedanken und Verabschiedung.

| **Telefoninterview**  **Glasgow Outcome Scale - Extended – Interview (GOSE)**  **+ modifizierte Rankin-Skala (mRS)**  Einwilligung des Patienten telefonisch: Datum:_____________ Uhrzeit:_______________  Einwilligung des gesetzlichen Vertreters telefonisch: Datum:_____________ Uhrzeit:_______________ | | | | | | | | |
| --- | --- | --- | --- | --- | --- | --- | --- | --- |
| ID:  Name des Patienten:_____________________ | | | | Datum der Befragung:_____________ | | | | |
| Geburtsdatum:____________ | | Datum der Verletzung:______ | | | | Geschlecht: M / W | | |
| Alter bei der Verletzung:______ | | | | Zeitraum seit der Verletzung:_______________ | | | | |
| Antwortender: | | Patient alleine ____ | | | | Angehöriger/ Freund/ Betreuer alleine ____ | | |
| Patient + Angehöriger/ Freund/ Betreuer ____ | | | | | | | | |
| Interviewer:_____________________________ | | | | | | | | |
| **BEWUSSTSEIN** | | | | | | | | |
| **1:** Ist die Person mit der Hirnverletzung fähig, einfache Anweisungen auszuführen oder zu sprechen? | | 1 = Nein **(VS)** | | | | 2 = Ja | | |
|  | | | | | | | | |
| Jeder, der einfache Anweisungen ausführen kann, einzelne Worte äußert oder auf andere Weise kommunizieren kann, gilt nicht länger als jemand, der sich in einem vegetativen Zustand (VS) befindet. Augenbewegungen gelten nicht als zuverlässiger Beweis für ein sinnvolles Antwortverhalten. | | | | | | | | |
| **UNABHÄNGIGKEIT IM HAUSHALT** | | | | | | | | |
| **2a:** Ist die Unterstützung einer anderen Person im Haushalt für die Ausführung einer Alltagsaktivität täglich erforderlich? | | 1 = Nein | | | | 2 = Ja | | |
|  | | | | | | | | |
| Bei der Antwort „Nein“ sollte er/sie in der Lage sein, wenn nötig, sich 24 Stunden zu Hause selbst zu versorgen, auch wenn er/sie sich normalerweise nicht ständig um sich selbst kümmern muss. Unabhängigkeit beinhaltet die Fähigkeit, folgende Tätigkeiten zu planen und auszuführen: sich waschen, unaufgefordert saubere Kleidung anziehen, sich selbst Essen zubereiten, Telefonanrufe entgegennehmen und kleinere Probleme im Haushalt lösen. Die Person sollte fähig sein, diese Tätigkeiten unaufgefordert und ohne daran erinnert zu werden auszuführen und in der Lage sein, nachts allein zu bleiben. | | | | | | | | |
| **2b:** Benötigt er/sie häufig Hilfe oder die fast ständige Anwesenheit einer weiteren Person zu Hause? | | 1 = Nein **(Upper SD)** | | | | 2 = Ja **(Lower SD)** | | |
|  | | | | | | | | |
| Bei der Antwort „Nein” sollte er/sie wenn nötig in der Lage sein, sich tagsüber bis zu 8 Stunden selbst zu versorgen, auch wenn er/sie sich normalerweise nicht ständig um sich selbst kümmern muss. | | | | | | | | |
| **2c:** War Unterstützung im Haushalt schon vor der Verletzung erforderlich? | | 1 = Nein | | | | 2 = Ja | | |
|  | | | | | | | | |
| **UNABHÄNGIGKEIT AUSSERHALB DES HAUSHALTS** | | | | | | | | |
| **3a:** Ist er/sie in der Lage, ohne Hilfe einzukaufen? | | 1 = Nein **(Upper SD)** | | | | 2 = Ja | |  |
|  | | | | | | | |  |
| Dies beinhaltet, dass er/sie in der Lage ist, zu planen, was einzukaufen ist, mit Geld eigenständig umzugehen und sich in der Öffentlichkeit angemessen zu verhalten. Er/sie muss nicht normalerweise einkaufen gehen, sollte aber prinzipiell dazu in der Lage sein. | | | | | | | |  |
| **3b:** Konnte er/sie vor der Verletzung ohne Hilfe einkaufen? | | 1 = Nein | | | | 2 = Ja | |  |
|  | | | | | | | |  |
| **4a:** Kann er/sie sich ohne Unterstützung in der näheren Umgebung Fahrten unternehmen? | | 1 = Nein **(Upper SD)** | | | | 2 = Ja | |  |
|  | | | | | | | |  |
| Er/sie kann Autofahren oder öffentliche Transportmittel benutzen, um sich fortzubewegen. Die Fähigkeit, ein Taxi zu benutzen, reicht aus, vorausgesetzt er/sie ist in der Lage, das Taxi selbst zu rufen und dem Fahrer die entsprechenden Anweisungen zu geben. | | | | | | | |  |
| **4b:** Konnte er/sie vor der Verletzung ohne fremde Hilfe Fahrten unternehmen? | | 1 = Nein | | | | 2 = Ja | |  |
|  | | | | | | | |  |
| **ARBEIT** | | | | | | | |  |
| **5a:** Ist seine/ihre Arbeitsfähigkeit im Vergleich zu früher unverändert? | | 1 = Nein | | | | 2 = Ja | |  |
|  | | | | | | | |  |
| Falls er/sie vorher berufstätig war, sollte seine/ihre jetzige Arbeitsfähigkeit auf dem gleichen Niveau sein. Falls er/sie vorher arbeitssuchend war, sollte die Verletzung seine/ihre Einstellungschancen oder das Niveau der Arbeit, für das er/sie qualifiziert ist, nicht beeinträchtigen. Wenn der Patient vor der Verletzung ein Student war, sollte die Verletzung keinen negativen Einfluss auf die Fortführung des Studiums haben. | | | | | | | |  |
| **5b:** Wie eingeschränkt ist die Person? | |  | | | |  | |  |
| a)  Verminderte Arbeitsfähigkeit | | | | 1 = a **(Upper MD)** | | | |  |
| b) Er/sie kann nur in einer Behindertenwerkstattarbeiten oder nur Arbeiten ohne Leistungsdruckausführen oder ist zurzeit nicht arbeitsfähig. | | | | 2 = b **(Lower MD)** | | | |  |
| **5c:** War er/sie vor der Verletzung berufstätig bzw. arbeitssuchend (Antwort „Ja”) oder keines von beidem (Antwort „Nein”)? | | 1 = Nein | | | | 2 = Ja | |  |
|  | | | | | | | |  |
| **SOZIALE KONTAKTE UND FREIZEITAKTIVITÄTEN** | | | | | | | |  |
| **6a:** Ist die Person weiterhin in der Lage, außerhalb ihres Haushalts regelmäßig soziale Aktivitäten oder Freizeitaktivitäten zu unternehmen? | | 1 = Nein | | | | 2 = Ja | |  |
|  | | | | | | | |  |
| Er/sie muss nicht in der Lage zu sein, sämtliche vorherigen Freizeitaktivitäten wieder aufzunehmen, er/sie sollte aber nicht durch physische oder geistige Beeinträchtigung daran gehindert werden. Falls er/sie die meisten Aktivitäten mangels Interesse oder Motivation aufgegeben hat, wird dies ebenfalls als Behinderung gewertet. | | | | | | | |  |
| **6b:** Wie groß ist das Ausmaß der Einschränkung der sozialen Kontakte und Freizeitaktivitäten? | | |  | | | |  |  |
| a) Nimmt etwas weniger teil: aber mindestens halb so oft wie vor der Verletzung. | 1 = a **(Lower GR)** | | | |  | |  |  |
| b) Nimmt deutlich weniger teil: weniger als halb so oft wie vor der Verletzung. | 2 = b **(Upper MD)** | | | |  | |  |  |
| c) Unfähig teilzunehmen: fast keine oder gar keine sozialen Kontakte oder Freizeitaktivitäten. | 3 = c **(Lower MD)** | | | |  | |  |  |
| **6c:** Hat er/sie vor der Verletzung regelmäßig außerhalb des Hauses soziale Aktivitäten und Freizeitaktivitäten unternommen? | 1 = Nein | | | | 2 = Ja | |  |  |
|  | | | | | | |  |  |
| **FAMILIE UND FREUNDE** | | | | | | |  |  |
| **7a:** Gab es psychologische Probleme, welche im Familien- oder Freundeskreis zu andauernden Zerwürfnissen geführt haben? | 1 = Nein | | | | 2 = Ja | |  |  |
|  | | | | | | |  |  |
| Typische post-traumatische Persönlichkeitsveränderungen nach der Verletzung: Jähzorn, Reizbarkeit, Ängstlichkeit, unsensibel anderen gegenüber, Stimmungsschwankungen, Depression, unvernünftiges oder kindisches Verhalten. | | | | | | |  |  |
| **7b:** Wie häufig waren Zerwürfnisse oder Spannungen? | | |  | | | |  |  |
| 1. Gelegentlich - weniger als einmal pro Woche. | 1 = a **(Lower GR)** | | | |  | |  |  |
| 1. Häufig – einmal pro Woche oder häufiger, aber noch erträglich. | 2 = b **(Upper MD)** | | | |  | |  |  |
| 1. Dauernd – täglich und unerträglich. | 3 = c **(Lower MD)** | | | |  | |  |  |
| **7c:** Gab es vor der Verletzung Probleme innerhalb der Familie oder mit Freunden? | 1 = Nein | | | | 2 = Ja | |  |  |
|  | | | | | | |  |  |
| Falls es schon vor der Verletzung Probleme gab, diese sich aber seither deutlich verschlimmert haben, ist die Frage 7c mit „Nein“ zu beantworten. | | | | | | |  |  |
| **WIEDEREINGLIEDERUNG INS NORMALE LEBEN** | | | | | | |  |  |
| **8a:** Bestehen gegenwärtig im Zusammenhang mit der Verletzung andere Probleme, die das tägliche Leben beeinflussen? | 1 = Nein **(Upper GR)** | | | | 2 = Ja **(Lower GR)** | |  |  |
|  | | | | | | |  |  |
| Andere typische Probleme, die nach Kopfverletzungen berichtet werden: Kopfschmerzen, Schwindelgefühle, Müdigkeit, Licht- oder Geräuschempfindlichkeit, Verlangsamung, Gedächtnisausfälle und Konzentrationsprobleme. | | | | | | |  |  |
| **8b:** Gab es ähnliche Probleme schon vor der Verletzung? | 1 = Nein | | | | 2 = Ja | |  |  |
|  | | | | | | |  |  |
| Falls es schon vor der Verletzung Probleme gab, diese sich aber seither deutlich verschlimmert haben, ist die Frage 8b mit „Nein“ zu beantworten. | | | | | | |  |  |

**Ergänzend zu mRS:**
(Wenn nicht schon bei bisherigen Fragen erwähnt)

1: Kann die Person ohne 1 = Nein 2 = Ja
fremde Hilfe gehen?

2: Benötigt die Person Hilfe 1 = Nein 2 = Ja
bei der Körperpflege?

3: Ist die Person ausschließlich 1 = Nein 2 = Ja
bettlägrig?

**Ergebnis GOS-E:**

1 Dead = Tot

2 Vegetative State (VS) = Vegetativer Zustand

3 Lower Severe Disability (Lower SD) = Schwere Behinderung: unteres Niveau (stark ausgeprägte schwere Behinderung)

4 Upper Severe Disability (Upper SD) = Schwere Behinderung: oberes Niveau (schwächer ausgeprägte schwere Behinderung)

5 Lower Moderate Disability (Lower MD) = Mittlere Behinderung: unteres Niveau (stärker ausgeprägte mittlere Behinderung)

6 Upper Moderate Disability (Upper MD) = Mittlere Behinderung: oberes Niveau (schwächer ausgeprägte mittlere Behinderung)

7 Lower Good Recovery (Lower GR) = Gute Erholung: unteres Niveau (weniger gute Erholung)

8 Upper Good Recovery (Upper GR) = Gute Erholung: oberes Niveau (gute Erholung)

**Ergebnis mRS:**

0 Keine Symptome.

1 Keine relevante Beeinträchtigung. Kann trotz gewisser Symptome Alltagsaktivitäten verrichten.

2 Leichte Beeinträchtigung. Kann sich ohne Hilfe versorgen, ist aber im Alltag eingeschränkt.

3 Mittelschwere Beeinträchtigung. Benötigt Hilfe im Alltag, kann aber ohne Hilfe gehen

4 Höhergradige Beeinträchtigung. Benötigt Hilfe bei der Körperpflege, kann nicht ohne Hilfe gehen.

5 Schwere Behinderung. bettlägerig, inkontinent, benötigt ständige pflegerische Hilfe.

6 Tod.
